# Secondary debulking for ovarian carcinoma relapse: The R-R dilemma – is the prognosis different for residual or recurrent disease?

**DOI:** 10.4274/jtgga.galenos.2019.2018.0165

**Published:** 2019-11-28

**Authors:** John D. Spiliotis, Christos Iavazzo, Nikolaos D. Kopanakis, Athina Christopoulou

**Affiliations:** 1 Department of Surgical Oncology and HIPEC, Athens Medical Centre, Athens, Greece; 2 Department of Surgical Oncology and HIPEC, European Interbalkan Medical Centre, Thessaloniki, Greece; 3 Clinic of Gynecological Oncology, Metaxa Cancer Hospital, Piraeus, Greece; 4 Clinic of Surgical Oncology, Metaxa Cancer Hospital, Piraeus, Greece; 5 Clinic of Medical Oncology, Saint Andrews Hospital, Patra, Greece

**Keywords:** HIPEC, ovarian carcinoma, relapse, residual, recurrence, management, survival, prognosis

## Abstract

**Objective:**

To analyze the kind of ovarian cancer relapse by separating residual from recurrent disease and correlating them with patient survival.

**Material and Methods:**

This was a retrospective study of 200 women with ovarian carcinoma relapse between 2005 and 2017.

**Results:**

The main sites of residual disease included the great omentum, epiploic appendices, liver round ligament, gallbladder, and cervical/vaginal stump. The median survival for women with residual disease treated with cytoreductive surgery (CRS) + hyperthermic intraperitoneal chemotherapy (HIPEC) + systemic chemotherapy was 38 months compared with the control group, which reached 23.8 months. The morbidity rates were 18% vs 7%, respectively, and the mortality rates were 2.5% vs 1.3%. The main sites of recurrent disease included the mesenterium, pelvic floor, diaphragm, and Glisson’s capsule. Women with recurrent disease treated with CRS + HIPEC + systemic chemotherapy had median survival rates of 26 months vs 16 months in the control group. The morbidity rates were 22% vs 15%, respectively, and the mortality rates were 3.3% vs 0%.

**Conclusion:**

Patients undergoing secondary debulking plus HIPEC for ovarian carcinoma relapse have a different prognosis when compared with patients with residual and recurrent disease. A different prognosis is presented in women undergoing secondary debulking plus HIPEC for ovarian carcinoma relapse when comparing patients with residual and recurrent disease.

## Introduction

Epithelial ovarian carcinoma (EOC) accounts for 2% of female cancer cases with high mortality rates and a five-year survival falling at 46%. Although the use of bevacizumab and poly adenosine diphosphate ribose polymerase inhibitors as well as an ultra-radical surgical approach to achieve zero residual disease were recently added in the current management, no satisfactory results can be achieved regarding progression-free survival (PFS) and overall survival (OS). However, ultra-radical debulking in combination with hyperthermic intraperitoneal chemotherapy (HIPEC) was revealed to be a safe and effective alternative approach. Around 70% of all women with ovarian carcinoma relapse after primary debulking and first-line chemotherapy.

The objective of our study was to discuss the possible differences in survival between residual and recurrent disease in patients with ovarian cancer presenting with disease relapse.

## Material and Methods

Two hundred patients with EOC relapse were retrospectively studied using our database. All patients with ovarian carcinoma relapse underwent surgery in three different hospitals by the same surgical group from 2005 to 2017.

**Figure 1:**
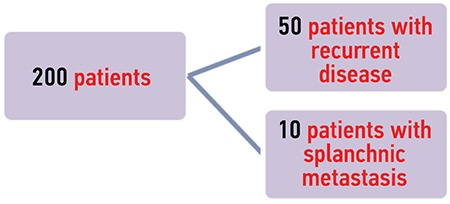
Flow chart of the patients’ cohort

During secondary cytoreduction, remaining abdominal disease after suboptimal or optimal primary or interval debulking was characterized as residual disease, and new disease found in patients who had primary or interval complete cytoreduction was considered as a recurrence. One hundred forty of 200 patients were detected as having residual disease compared with 50/200 with recurrent disease and 10/200 with splanchnic metastases (Figure 1). 

**Figure 2:**
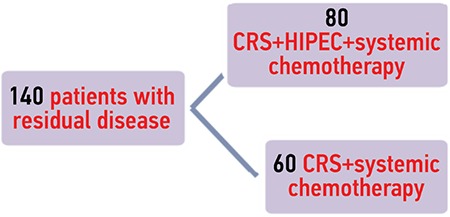
Division of patients with residual disease CRS: Cytoreductive surgery; HIPEC: Hyperthermic intraperitoneal chemotherapy

**Figure 3:**
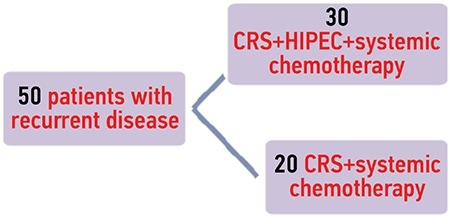
Division of patients with recurrent disease CRS: Cytoreductive surgery; HIPEC: Hyperthermic intraperitoneal chemotherapy

Both groups of patients with recurrent and residual disease were divided in two subgroups: CRS + HIPEC followed by systemic chemotherapy, and a second subgroup receiving CRS + systemic chemotherapy alone. The ten patients with splanchnic metastases received systemic chemotherapy (Figure 2, 3).

## Results

The mean age of the patients was 69 (range, 42-83) years. The mean body mass index was 31 (range, 24-43) kg/m^2^. Thirty-four patients had a family history of ovarian cancer. No information was available regarding their BRCA status. All patients had initially received 6 cycles of carboplatin and taxol. The platinum-free interval was more than 6 months in all cases ranging from 10 months to 22 months. A difference was found between the sites of recurrent and residual disease. The main sites of residual disease included the great omentum (67%), epiploic appendices (33%), liver round ligament (55%), gallbladder (33%), and the cervical/vaginal stump (30%). The recurrent disease sites in the residual disease group were at the same sites seen in the primary surgery. The median preoperative peritoneal cancer index (PCI) was 18 and we achieved complete cytoreduction in 75%; 20% of the women experienced grade 3 and 4 complications. The median OS for women with residual disease treated with CRS + HIPEC + systemic chemotherapy was 38 months compared with the control group, which reached 23.8 months (Table 1). In this group of patients, the morbidity rates were 18% vs 7%, respectively, and the mortality rates were 2.5% vs 1.3%. The main sites of recurrent disease included the mesenterium (50%), pelvic floor (40%), diaphragm (60%), and Glisson’s capsule (40%). The median preoperative PCI was 22 and we achieved complete cytoreduction in 64%; 14% of the patients experienced grade 3 and 4 complications. In the recurrent disease group, the median OS rates reached 26 and 16 months, respectively (Table 2). In this group of patients, the morbidity rates were 22% vs 15%, respectively, and the mortality rates were 3.3% vs 0%.

## Discussion

Recurrent ovarian cancer is treatable but rarely curable. The recurrence rates depend on the stage at diagnosis reaching 10%, 30%, 70-90%, and 90-95% for stages I to IV, respectively (1). One of the main factors affecting the patient’s risk of recurrence is the completeness of primary/interval debulking. The majority of women with ovarian cancer have recurrence in the peritoneal cavity independent of the primary/interval debulking extent and/or type of chemotherapy (2). Rose et al. (3) proposed a nomogram for predicting individual survival after ovarian cancer recurrence, which included time to recurrence after initial chemotherapy, clear cell or mucinous histology, performance status, stage IV disease, and age. A recent retrospective study revealed that peritoneal recurrence was found in 75% of patients with advanced disease, and relapse was found at both treated and untreated sites. Nodal relapse was found in 38% of all cases, and isolated distant metastases were identified in 8% of patients (4). According to Ushijama (1), around 55% of women have recurrence at the primary site and the rest present with distant metastases including retroperitoneal nodes, liver or spleen, brain, and bone. In our study, the main areas of relapse included the great omentum, epiploic appendices, liver round ligament, gallbladder, and the cervical/vaginal stump in the residual disease group compared with the mesenterium, pelvic floor, diaphragm, and Glisson’s capsule in the recurrent disease group. Women with recurrent ovarian cancer may be eligible for secondary cytoreduction (1).

The DESKTOP trial suggested the main selection criteria of operability for patients with recurrent ovarian cancer, including good performance status, absence or small volume of ascites at recurrence, and completeness of primary surgery (5). Recently, DESKTOP III revealed that secondary cytoreduction led to improved PFS (19.6 months vs 14 months) compared with second-line chemotherapy in 407 relapsed patients after a progression-free interval period of more than 6 months as well as a positive the Arbeitsgemeinschaft Gynaekologische Onkologie-score performance status, Eastern Cooperative Oncology Group 0, ascetic volume of less than 500 mL, and zero residual tumor at initial debulking (6). Regarding OS rates, the results remain immature and are not yet published (6). Another study proposed that the main predictors for complete cytoreduction in women undergoing secondary cytoreduction included stage of disease, complete primary/interval debulking surgery, PFS, CA125 values and presence of ascites at recurrence (7). Based on the above, Zang et al. (8) suggested a prognostic model to predict survival benefit from secondary debulking including four parameters (progression-free interval, presence of ascitic fluid at recurrence, extent of recurrent disease, and completeness of secondary cytoreduction based on the residual disease. More specifically, the median survival after secondary debulking for women with progression-free intervals >23.1 months was 45.0 months compared with 21.0 months in women with progression-free intervals of <23.1 months. The cut-off level of CA125 at recurrence was found as 251.0 U mL^-1^. Median survival was found as 43.9 months in women with local disease compared with 20.0 months in patients with multiple areas of recurrence (8). Zero residual disease after secondary cytoreduction was the strongest prognostic factor. More specifically, the median survival was 57.7 months in women achieving R0 during secondary cytoreduction compared with 27.0 months in the R1 group, and 15.6 months in the R2 group (8,9). Furthermore, Laga et al. (10) confirmed that DESKTOP score and the Tian model were the main predictors of candidate selection for complete secondary cytoreduction. However, in their study, 61% and 70% of the patients were debulked to R0 independently of the negative preoperative scores. For this reason, they suggested that other anatomic and metabolic imaging criteria should be evaluated to recognize eligible patients for HIPEC plus secondary cytoreduction (10).

HIPEC following secondary cytoreduction is an alternative approach for patients with recurrent ovarian disease. Harter et al. (5) concluded that “HIPEC remains experimental in ovarian cancer patients but it can be used inside prospective controlled trials”. A recent meta-analysis showed better OS rates for patients with recurrent ovarian cancer when adding HIPEC to secondary cytoreduction and traditional chemotherapy. Additionally, a positive correlation between completeness of debulking and survival was found. In the same analysis, morbidity and mortality rates were similar (11).

It should be highlighted that in high-volume centers with HIPEC specialists, morbidity and mortality has drastically improved (12,13). The published results from our center showed that women with advanced ovarian carcinoma recurrence had a mean survival benefit of around 13.3 months when HIPEC is offered (26.7 months vs 13.4 months in the non-HIPEC group) (14). Hotouras et al. (15) showed that in women with ovarian carcinoma recurrence undergoing debulking plus HIPEC administration, the OS ranged between 26.7 and 35 months, with PFS varying between 8.5 and 48 months. The role of HIPEC in patients with ovarian cancer was recently confirmed in a randomized controlled trial that highlighted a better PFS (15 months vs 11 months) as well as OS (46 months vs 34 months) in patients with stage III EOC undergoing interval cytoreduction plus HIPEC administration (16). The results of other randomized trials in the field are awaited.

The questions raised by our study related to whether disease recurrence refers to relapse or residual disease post initial surgery, and whether secondary cytoreduction followed by HIPEC has a different effect on PFS and OS in the two different groups. This was actually confirmed from our results because the median survival for women with residual disease treated with CRS + HIPEC + systemic chemotherapy was 38 months compared with the control group, which reached 23.8 months. In addition, patients who presented with recurrent disease had median survival rates of 26 months and 16 months, respectively. To summarize, the addition of HIPEC improves survival rates in both patients with residual as well as recurrent disease, and such rates were obviously better in the residual tumor group compared with the recurrent disease group. Such findings also highlight the need of major cytoreductive effort/ultra-radical surgery at the moment of primary/interval cytoreduction.

This study has some limitations that have to be addressed, including the small patient population and the retrospective nature of the study. It is a well-known fact that maximal and optimal cytoreduction have better prognosis than suboptimal debulking. One hundred forty patients had residual disease in our study. This number could be considered quite high, but we should clarify that all these patients were referred to our group for further management in our tertiary centers after undergoing surgery either by non-subspecialists or in cases where neoadjuvant chemotherapy had not been considered an option prior to primary debulking. Unfortunately, because the majority of patients were initially treated by non-subspecialists, we are unable to subdivide optimal and suboptimal cytoreduction categories in the residual disease group.

Our retrospective study shows that HIPEC improves survival rates in both patients with residual as well as recurrent disease. Better survival rates were found in women with residual disease treated with HIPEC – rates that were are actually longer compared with the recurrent group. Prospective randomized multicenter studies are essential to further empower our findings.

## Figures and Tables

**Table 1:**
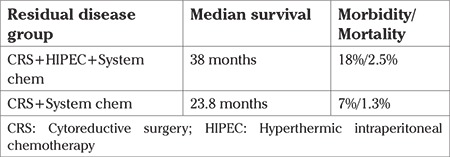
Survival, morbidity and mortality rates in patients with residual disease

**Table 2:**
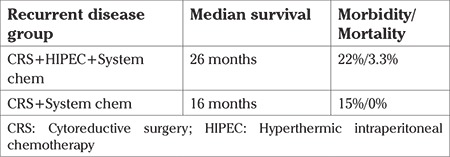
Survival, morbidity and mortality rates in patients with recurrent disease
